# Self-Eating: Friend or Foe? The Emerging Role of Autophagy in Idiopathic Pulmonary Fibrosis

**DOI:** 10.1155/2013/420497

**Published:** 2013-04-10

**Authors:** George A. Margaritopoulos, Eliza Tsitoura, Nikos Tzanakis, Demetrios A. Spandidos, Nikos M. Siafakas, George Sourvinos, Katerina M. Antoniou

**Affiliations:** ^1^Interstitial Lung Disease Unit, University Hospital of Heraklion, 71110 Heraklion, Crete, Greece; ^2^Laboratory of Virology, Medical School, University of Crete, 71110 Heraklion, Crete, Greece

## Abstract

Idiopathic pulmonary fibrosis is the most common and severe form of idiopathic interstitial pneumonias. Despite an exponential increase in our understanding of potentially important mediators and mechanisms, the pathogenesis remains elusive, and little therapeutic progress has been made in the last few years. Mortality in 3–5 years is still 50%. Autophagy, a highly conserved homeostatic mechanism necessary for cell survival, has been recently implicated in the pathogenesis of pulmonary disorders. In this paper we aim to highlight some key issues regarding the process of autophagy and its possible association with the pathogenesis of idiopathic pulmonary fibrosis.

## 1. Introduction

Idiopathic pulmonary fibrosis (IPF) is a chronic and progressive fibrosing interstitial pneumonia of unknown cause whose pathogenesis, despite recent advances, is still not fully understood [1]. It is currently believed that IPF is the result of repeated injuries in different sites of the alveolar epithelium with dysregulation of cellular homeostasis followed by aberrant wound healing and inadequate repair of the epithelial damage. Median survival from the time of diagnosis is approximately 3 years, and surprisingly the mortality rate exceeds that of many cancers [2]. Lung transplantation is the only therapeutic approach which can affect survival, but it is limited to highly selected patients. However, recent clinical trials have yielded encouraging results regarding the use of pirfenidone and N-acetyl-cysteine in mild to moderate disease [3, 4].

In view of the limited current knowledge and poor survival of the disease, the need for an elucidation of the pathogenesis becomes imperative. Recently, the process of autophagy, a term derived from the Greek and meaning “self-eating,” has been implicated in the pathogenetic pathway of IPF [5, 6]. Autophagy is a highly conserved homeostatic mechanism by which cells transport damaged proteins and organelles such as mitochondria to lysosomes for degradation in both health and disease conditions. It contributes to cellular homeostasis by (a) providing an alternative source of metabolic fuel, (b) removing damaged cellular components which are toxic to the cell such as dysfunctional mitochondria or aggregated proteins, and (c) promoting cell death [7]. 

In previous studies, the term of selective autophagy has been introduced in order to underline the selective targeting of cargoes for degradation. Mitophagy refers to selective digestion of mitochondria, and xenophagy refers to selective degradation of invading pathogens and bacteria [8, 9]. The selective degradation of protein aggregates is regulated by p62, a cytosolic chaperone protein.

 Most of the current knowledge regarding the role of autophagy in cell homeostasis was obtained by studies in yeast and mice, whereas its role in human diseases has only been slightly investigated and appears to be highly pleiotropic as it may either represent an adoptive prosurvival response or, if deregulated, promote cell death and morbidity. Autophagic cell death differs from apoptotic cell-death; the former does not involve caspase activation. Nonetheless, the two processes can coexist [10].

In this paper, we aim to highlight the current knowledge regarding the process of autophagy and its role in various pulmonary disorders with a particular focus on IPF. 

## 2. Classes and Regulation of Autophagy

There are three mechanisms by which autophagy can occur. Microautophagy involves a nonselective surrounding of cytoplasmic components directly by lysosomal membranes. The chaperone-mediated autophagy involves a selective transport to lysosomes of cargoes that contain a specific pentapeptide motif (KFERQ). Macroautophagy is characterized by the presence of autophagosome, a double-membraned vesicle that surrounds the damaged component to be degraded following fusion with endosomes and lysosomes. Chaperone mediated autophagy and microautophagy are less studied whereas macroautophagy (referred as autophagy hereafter) is the best studied mechanism since autophagosomes are easy to detect with fluorescence and electron microscopy [11]. The process consists of in four distinct steps: (a) the formation of an isolation membrane, (b) the formation of an autophagosome with engulfment of the cargo, (c) the fusion of the autophagosome with a lysosome, and (d) the degradation of the cargo by lysosomal enzymes with regeneration of metabolic precursor molecules to be used for anabolic pathways [7]. 

A series of autophagy-related genes (Atg) are involved in the regulation of the process. Beclin-1, an interacting protein, in complex with class III phosphatidylinositol 3-kinase (PI3 K) and Atg14 acts as a major positive regulator of autophagy [12]. The rapamycin-sensitive mammalian target of rapamycin (mTOR)/class I PI3 K pathway acts as a major negative regulator of autophagy [13, 14]. Autophagosome formation requires two ubiquitin-like conjucation systems: the Atg5–Atg12 conjucation system and the Atg8 (microtubule-associated protein-1 light chain [LC] (3) conjugation system by which LC3 is converted from LC3-I (free form) to LC3 II (conjugated to phosphaditylethanolamine form) a step which is considered critical for autophagosome formation [15–17]. The fusion of autophagosome with lysosome requires the involvement of a GTPase termed Rab-7 and lysosome-mediated membrane protein (LAMP) −1 and −2.

Autophagy can be measured with various methods, and each one has its advantages and limitations. Electron microscopy can visualize early-stage autophagosomes but is less sensitive for the visualization of late-stage autophagosomes [18]. Fluorescence-based methods such as the use of green fluorescent protein (GFP)-LC3 are also used. They are based on the fact that when autophagy is induced and LC3b becomes part of the newly formatted autophagosome, the GFP-LC3 changes its cellular localization from a diffuse cytosolic pattern to a punctuate pattern [19]. Western blot analysis has demonstrated that LC3b-II correlates well with the number of autophagosomes and, thus, with autophagic activity [20]. Western blot analysis of p62, a cytosolic chaperone protein that has an LC3b binding domain [21], can also be used as an increase of p62 levels is associated with the reduced autophagic activity [22]. These methods have the disadvantage of evaluating autophagy in a certain time point (i.e., static measures) and may not reflect the autophagic activity properly as an increase of either the number of autophagosomes or the levels of LC3b-II may be due to the enhanced induction of autophagosome formation or to inhibited fusion with lysosomes which in reality means low autophagic flux. In order to distinguish between these two options, autophagic flux assays are used (i.e., dynamic measures). In cell cultures, LC3b-II levels are measured with Western blot in presence and absence of inhibitors of autophagic degradation such as chloroquine, leupeptin, and bafilomycin-A [23]. Another pitfall regarding the measurement of autophagy in lung diseases is that the process is cell-dependent as it was shown in COPD [24, 25] where it is enhanced in the epithelium and impaired in alveolar macrophages. Thus, the study of autophagy in samples such as whole lung homogenates may not represent what happens in a specific subset of cells. A combination of static and dynamic methods is currently recommended with a careful definition of the type of cell in which autophagy is being measured [7].

## 3. Autophagy in Pulmonary Diseases

Autophagy has been implicated in the pathogenesis of several pulmonary diseases (Table 1) and represents a potential therapeutic target in current and future clinical trials (Table 2) [26]. Chronic obstructive pulmonary disease (COPD) is the best studied lung disorder regarding the role of autophagy and represents an example of the cell-specific role of autophagy in the context of the same disease. In lung biopsy samples, it was observed that the number of autophagosomes as well as the levels of LC3b-II and other autophagy-related proteins was increased and moreover was correlated with disease severity [27]. Similar results were obtained in lung epithelial cell lines and fibroblasts exposed to cigarette smoking extract (CSE) which is the primary causative agent of COPD [27, 28]. Genetic depletion of Beclin-1 and LC3b decreased cell death in exposed cells, and mice deficient in LC3b did not develop emphysema after exposure to CSE [24, 27, 29]. On the other hand, in alveolar macrophages, autophagic flux was impaired, and the effect of smoking was further supported by the fact that similar changes were observed in alveolar macrophages of non smokers exposed to cigarette smoking [25]. Therefore, in conclusion, enhanced autophagy in epithelial cells has a deleterious effect by promoting cell death and developing of emphysema, and impaired autophagy in alveolar macrophages contributes to the deficient transport of bacteria to lysosome, namely, xenophagy, and to the higher propensity of COPD patients to bacterial acute respiratory infections which lead to acute exacerbations. 

Cystic fibrosis (CF) is a disease characterized by mutations of the cystic fibrosis transmembrane conductance regulator (CFTR) gene which is associated to intracellular accumulation of misfolded proteins that are supposed to be cleared by autophagy. It has been observed that in CF human epithelial cell, mutant CFTR proteins impair autophagosome formation via depletion of Beclin-1 [30] and increase of reactive oxygen species (ROS) and transglutaminase-2 production which contributes to the excessive inflammation seen in CF. Restoring Beclin-1 restores autophagy measured by LCEb-II and p62 levels and GFP-LC3 puncta and leads to increased clearance of protein aggregates and marked reduction of inflammation in a mouse model of CF [30]. Interestingly, treatment with N-acetylcysteine (NAC) also has beneficial effects suggesting a possible implication of antioxidants in the therapy of CF. Infection by *Burkholderia cenocepacia* is potentially lethal in patients with CF, and it was observed that it is associated with downregulation of several autophagic genes in alveolar macrophages [31]. Treatment with rapamycin induced autophagy and reduced lung inflammation. 

 Alveolar macrophages provide the first line of defense against invading microbes. Infection by *Mycobacterium tuberculosis *is also associated with inhibition of the formation of autophagosomes which inhibits mycobacterial killing by alveolar macrophages [32]. Stimulation of alveolar macrophages with interferon-*γ* (IFN-*γ*) upregulates a GTPase, namely, IRGM-1, and activates autophagy [33]. Induction of autophagy by rapamycin also enhances mycobacterial killing [34]. 

Autophagy seems to be implicated in the development of pulmonary hypertension (PH). In lung samples of human PH and lung vasculature, there was an increased expression of LC3b and GFP-LC3 puncta formation, a marker of autophagosome formation in GFP-LC3-transfected endothelial cells. When mice genetically deficient in LC3b were exposed to chronic hypoxia, they demonstrated an evidence of increased pulmonary hypertension compared to wild-type mice suggesting a protective role of autophagy by limiting hypoxia-dependent vascular cell proliferation [35].

Tuberous sclerosis complex (TSC) is an autosomal dominant tumor suppressor gene syndrome caused by germline mutations in the TSC1 or TSC2 genes. Patients with TSC have multisystem manifestations such as neurologic disease, benign tumors in multiple organs, and pulmonary lymphangioleiomyomatosis (LAM). Genetic and pharmacologic autophagy inhibition blocks tumorigenesis in xenograft and spontaneous models of TSC [36] and hence may represent a potential therapeutic target for TSC.

Autophagy has functional implications in the pathogenesis of cancer, but only few studies have been performed specifically in lung cancer, and the role of autophagy in response to chemotherapeutic agents using cultured human lung A549 adenocarcinoma cells [37, 38] has been evaluated. Interestingly, Nelfinavir, an HIV protease inhibitor, exerted pleiotropic biochemical and cellular effects that included induction of endoplasmic reticulum (ER) stress, autophagy, and apoptosis *in vitro* and *in vivo* and exhibited antiproliferative activity in lung cancer cells [39]. 

## 4. Autophagy and IPF: Indirect Pathogenetic Links

A growing body of evidence suggests that there may be a pathogenetic link between IPF and autophagy. Oxidative stress, endoplasmic reticulum (ER) stress, and hypoxia, all mechanisms that participate in the pathogenesis of IPF [40–42], are well-known inducers of autophagy [43–45]. On the other hand, viral infections, which have also been hypothesized to favor the development of fibrosis, seem to have an inhibitory effect on autophagy.

ER is an organelle that serves general functions such as facilitation of protein folding and transport of newly synthesized proteins. Oxidative stress, disturbances in calcium regulation, glucose deprivation, and viral infection can cause ER stress which leads to increase of the unfolded proteins [46]. In neuroblastoma cells, ER stress induces autophagy and is associated with cell survival [47]. However, ER-induced autophagy may be cell specific because it was seen that in colon and prostate cancer cell lines activation of autophagy by chemical inducers reduces cell death, whereas in normal human colon cells and in nontransformed embryonic fibroblasts, it contributes to cell-death [48]. In lung epithelial fibroblast cell lines, autophagy induced by chemicals resulted in increased accumulation of LC3-II and activation of unfolded protein response, a compensatory mechanism to ER stress [49] suggesting a possible protective role of autophagy. 

It is now accepted that IPF is the result of multiple injuries in the epithelium which leads to early death of type II alveolar epithelial cells (AECs II) and aberrant wound healing. In cases of familial IPF, it was shown that mutations of the surfactant protein-C gene (SFTRC) lead to accumulation of misfolded proteins, induction of ER stress, and apoptosis of AEC II [50–53]. In sporadic IPF and regardless the absence of mutations, an increase of ER stress and apoptotic markers in AEC II was also observed [54]. Recently, it was also observed that ER stress is implicated in the differentiation of fibroblast into myofibroblast which is considered a key event in the pathogenesis of IPF [40]. 

Recent evidence suggests that oxidative stress, defined as an imbalance of the generation of ROS in excess of the capacity to neutralize them, promotes autophagy. According to a hypothesis [55], mild levels of oxidative stress activate autophagy in order to eliminate the damaged organelles and thus to promote cell survival. On the other hand, acute or persistent oxidative stress, which has been hypothesized in the pathogenesis of IPF, leads to an increase of intracellular ROS, damage of the lysosomal membrane with intracellular release of potent hydrolases, dysregulation of autophagy with perpetuation of the oxidative injury, and initiation of a vicious circle that leads to apoptotic cell death. In transformed and cancer cell lines treatment with hydrogen peroxide (H_2_O_2_) induced autophagy and promoted caspase-independent cell death, whereas knockdown of specific autophagic genes with small interfering RNAs (siRNAs) prevented H_2_O_2_-induced autophagic cell death [56]. Starvation, which is a known inducer of autophagy, is associated with an increase of intracellular ROS and leads to autophagosome formation and autophagic degradation in CHO and HeLa cells [57]. In the former scenario [56], ROS-induced autophagy led to cell death, whereas in the latter [57] it represented a mechanism which is essential for cell survival. In the model of human bronchial epithelial cells treated with cigarette smoke extract, an increase of intracellular ROS was observed, and the activation of autophagy had a deleterious effect promoting the death of epithelial cells [26]. 

ROS and markers of oxidative stress are evident in IPF, and levels of ROS are negatively correlated with lung function [58–60]. The overproduction of ROS may cause lung injury and promote a tissue microenvironment which favors fibrosis over regeneration. Glutathione, an antioxidant agent, has been found to be decreased in IPF [61]. NAC is capable of stimulating glutathione synthesis, increasing the intra- and extracellular levels, and thereby partially restoring glutathione levels [62, 63]. NAC has been found to have favourable effects on the lung function of patients with IPF and mainly in those with less progressed disease [4, 64]. However, results of a recent trial showed some conflicting results which need further careful investigation [65]. 

Autophagy is sensitive to oxygen tension, and hypoxia inducible factor 1-*α* (HIF 1-*α*) has been implicated as a regulator of autophagy and of turnover of damaged mitochondria under hypoxic condition. HIF 1-*α* target gene, namely, Bcl-2/adenovirus E1B 19 kDa—interacting protein-3 (BNIP3), also regulates hypoxia-induced autophagy [45, 66]. However, even in the case of hypoxia, the dual role of autophagy has been emerged as in another study, it was observed that prolonged hypoxia induces autophagic cell death through a BNIP3 dependent mechanism [67]. 

It is now believed that hypoxia can lead to alveolar epithelial cell apoptosis initiating the cascade of fibrogenesis. In fact, it was observed in both animal models of bleomycin-induced fibrosis and in lung tissues of IPF patients that HIF 1-*α* is overexpressed and may exert its role in early stages of fibrogenesis as it was localized in areas of active fibrosis and in normal areas of IPF lung but not within the fibroblastic foci which represent areas of established fibrosis [42].

Autophagy, as part of the host defence system, has been targeted by viral proteins through the evolution of mechanisms of virus escape. The alpha-herpesvirus HSV-1 inhibits autophagy (i.e., xenophagy) through the actions of ICP34.5 and US11 proteins. ICP34.5 protein directly binds Beclin1 leading to the inhibition of the autophagosome formation [68]. As part of the intrinsic antiviral response, infected cells block protein synthesis through the PKR-mediated phosphorylation of eIF2a translation initiation factor, a process that also leads to the upregulation of autophagy [69]. The ICP34.5 and US11 proteins inhibit the phosphorylation of eIF2a at temporally distinct phases of HSV-1 infection, thereby releasing the block to protein synthesis and subsequently inhibiting the induction of autophagy [70, 71]. Similar to alpha-herpesviruses, beta-herpesviruses like hCMV are extremely efficient in blocking autophagosome formation through the TRS1 viral protein which directly interacts and inhibits Beclin1 [72]. Gamma-herpesviruses seem to employ a different mechanism for the inhibition of autophagy which relies on the acquisition of cellular homologues of Bcl-2 protein including BHRF1 and BALF-1 of EBV, Orf16 of Kaposi's sarcoma-associated herpesvirus (KSHV), and M11 of murine *γ*-herpesvirus 68 (*γ*-HV68) [73]. Bcl-2 protein apart from its antiapoptotic role acts as a potent inhibitor of autophagy through Beclin binding. Bcl-2 is regulated through JNK phosphorylation, upon which Bcl-2 is released from Beclin-1 allowing for the activation of autophagy. The viral Bcl-2 analogues expressed by several gamma-herpesviruses lack the JNK phosphorylation domain, thereby escaping JNK regulation and acting as dominant inhibitors of autophagy.

Several studies have suggested a link between IPF and occult viral infections in the lung, including herpesviruses, adenovirus, hepatitis C, and Torque teno virus. It has been hypothesized that these viruses may represent injurious agents in the context of the “multiple hits” hypothesis. The Epstein-Barr virus (EBV) has been detected in both familial and sporadic IPF [74], and EBV protein and DNA expression have been found in IPF lung tissues [75, 76]. EBV replication has been demonstrated in type II alveolar epithelial cells, and EBV latent membrane protein 1 (LMP-1) expression was detected in the alveolar epithelium in IPF patients, findings that were associated with poor prognosis [77]. Moreover, from the clinical point of view, antiviral treatment has been reported to stabilise the course of IPF [74]. Recently, our group has detected the presence of HSV-1 in patients with fibrotic idiopathic interstitial pneumonias, since the virus presented similar incidence in two different biological samples, tissue, and bronchoalveolar lavage fluid. We have also found that the presence of HSV-1 can enhance fibrosis by inducing the transcription of molecular pathways which promote fibrotic, angiogenetic, wound healing, and innate immunity processes, suggesting a probable role of infectious factors in the pathogenesis of lung fibrosis [78]. Proof of concept experiments of the involvement of herpesviruses in lung fibrosis come from experimental models of pulmonary fibrosis with the MHV-68 murine gamma-herpesvirus. Importantly, experimentally established pulmonary latent infection of mice with MHV-68 could confer higher susceptibility to bleomycin or FITC-induced fibrosis [79] in comparison to the uninfected control mice, thereby supporting a multiple/recurrent hit hypothesis where the herpesvirus presence alters the lung microenvironment and acts as a cofactor in experimentally induced models of pulmonary fibrosis.

## 5. Autophagy in IPF 

Recently, Mi et al. have shown that IL17A, a cytokine that induces production of collagen and promotes epithelial-mesenchymal transition (EMT) through a transforming-growth-factor-*β*1- (TGF-*β*1-) dependent mechanism, inhibits autophagy in mouse epithelial cells [80]. Moreover, they observed that in the murine model of BLM-induced fibrosis, antagonism of IL17A activated autophagy, decreased the production of collagen, attenuated fibrosis and increased survival. This protective effect was abolished after blocking autophagy with 3-methyladenine (3-MA). 

Patel et al. studied markers of autophagic activity and concluded that autophagy is not induced in human IPF lungs [5]. More in detail, they observed that the levels of LC3-II were lower and the levels of p62 were higher in IPF lungs compared to controls. Moreover, they observed a decrease in the number of autophagosomes with electron microscope in IPF lungs. In order to provide a plausible answer, they used fibroblast cell lines and showed that TGF-*β*1, a profibrotic cytokine which is overexpressed in IPF, inhibits autophagy. Silencing of LC3 and beclin-1 genes and, hence, inhibiting autophagy enhanced the expression of fibronectin and *α*-smooth cell actin, a marker of myofibroblast which is a key cell in the process of fibrogenesis. In the BLM model, they observed that treatment with rapamycin enhanced autophagy and protected from fibrosis. Based on these observations, the authors concluded that autophagy protects against the development of fibrosis.

A growing body of evidence at both clinical and biological level suggests that IPF is a disease of aging characterised by premature age-related changes in alveolar epithelial cells [81]. It is also accepted that autophagy functions less as tissue ages due to insufficient formation of autophagosomes or to deficient elimination after fusion with lysosomes [82]. Recently, Araya et al. also attempted to clarify the role of autophagy in IPF [6]. They suggested that insufficient autophagy leads to epithelial cell senescence as they observed an increased expression of p62 and ubiquitinated proteins, both markers of decreased autophagy as well as an increased expression of p21 which is a marker of cellular senescence. On the other hand and in agreement with previous observations of cell-specific effect of autophagy, they showed that insufficient autophagy in lung fibroblasts leads to the differentiation to myofibroblasts without any effect on their senescence and to increased production of extracellular matrix which are critical steps in the fibrogenetic process.

## 6. Conclusion

Over the last decade, there has been an explosion in the research field regarding the possible mechanisms involved in the pathogenesis of IPF and clinical trials in order to find a therapeutic agent able, at least, to stabilise the course of the disease. Despite all these efforts, pathogenesis of IPF is still not fully understood. A universally accepted therapeutic agent has not yet been found. Recently, it has been suggested that there should be an “oncologic” approach in the pathogenesis of IPF. According to this hypothesis, multiple pathways may be involved simultaneously in the pathogenesis of IPF, and future therapeutic approaches should target these pathways simultaneously [83]. Moreover, this hypothesis has been strengthened by the observation that there are certain similarities between IPF and lung cancer biology [2]. Autophagy has been recently implicated in the pathogenesis of lung diseases, and only few studies exist in the field of IPF, and thus its role is rather obscure. Therefore, more studies are needed in order to clarify the role of autophagy in IPF and in order to develop novel therapeutic approaches.

## Figures and Tables

**Table 1:** Autophagy and pulmonary diseases.

Study	Model	Disease	Stimulus/effect
Chen et al. 2008 [24]	*in vivo/in vitro *	COPD	CS induces autophagy in epithelial cells.
Monick et al. 2010 [25]	*in vitro *	COPD	CS-induced cell death and emphysema are regulated by LC3*β* activity.
Chen et al. 2010 [29]	*in vivo/in vitro *	COPD	CS decreases autophagy in alveolar macrophages.
Luciani et al. 2010 [30]	*in vivo/in vitro *	CF	Defective autophagy in CF
Abdulrahman et al. 2011 [31]	*in vivo/in vitro *	CF	Treatment with rapamycin induces autophagy and reduces the burden of *B*. *cenocepacia*.
Singh et al. 2006 [33]	*in vitro *	MTB infection	Induction of autophagy by IFN*γ* or rapamycin enhances mycobacteria killing.
Gutierez et al. 2004 [34]	*in vitro *	MTB infection	Induction of autophagy by IFN*γ* eliminates mycobacteria through an IRGM mechanism.
Lee et al. 2011 [35]	*in vivo/in vitro *	PH	Autophagy protects against hypoxia-induced PH.
Parhitko et al. 2011 [36]	*in vivo/in vitro *	TSC	Genetic-pharmacologic inhibition of autophagy blocks autophagy in TSC.
Gills et al. 2007 [39]	*in vivo/in vitro *	Lung cancer	Nelfinavir activates autophagy and exhibits antiproliferative activity in lung cancer.
Mi et al. 2011 [80]	*in vivo/in vitro *	PF	Antagonism of IL-17A induces autophagy and protects against fibrosis.
Patel et al. 2012 [5]	*in vivo/in vitro *	IPF	TGF-*β*1 inhibits autophagy in lung fibrobasts. Rapamycin induces autophagy in the BLM model and reduces the degree of fibrosis.
Araya et al. 2013 [6]	*in vitro *	IPF	Insufficient autophagy promotes myofibroblast differentiation and collagen deposition.

COPD: chronic obstructive pulmonary disease, CS: cigarette smoking, CF: cystic fibrosis, MTB: mycobacterium tuberculosis, IFN*γ*: interferon *γ*, PH: pulmonary hypertension, TSC: tuberous sclerosis complex, PF: pulmonary fibrosis, IPF: idiopathic pulmonary fibrosis, TGF-*β*1: transforming growth factor-*β*1, BLM: bleomycin.

**Table 2:** Clinical trials targeting autophagy in pulmonary diseases.

Identifier	Condition	Intervention	Phase	Status
NCT00969306	SCLC	Chloroquine, A-CQ100	Phase 1	Not yet recruiting
NCT00933803	NSCLC	Paclitaxel Carboplatin Hydroxychloroquine Bevacizumab	Phase 1 Phase 2	Active Not recruiting
NCT01649947	NSCLC Recurrent NSCLC	Paclitaxel Carboplatin Hydroxychloroquine Bevacizumab	Phase 2	Recruiting
NCT00728845	Recurrent Advanced NSCLC	Bevacizumab Carboplatin Hydroxychloroquine Paclitaxel	Phase 1 Phase 2	Terminated
NCT01687179	LAM	Sirolimus and hydroxychloroquine	Phase 1	Recruiting

SCLC: small-cell lung cancer, NSCLC: non-small cell lung cancer, LAM: lymphangioleiomyomatosis.
